# Feruloylated Arabinoxylans from Nixtamalized Maize Bran Byproduct: A Functional Ingredient in Frankfurter Sausages

**DOI:** 10.3390/molecules24112056

**Published:** 2019-05-30

**Authors:** Daniela D. Herrera-Balandrano, Juan G. Báez-González, Elizabeth Carvajal-Millán, Gerardo Méndez-Zamora, Vania Urías-Orona, Carlos A. Amaya-Guerra, Guillermo Niño-Medina

**Affiliations:** 1 Departamento de Alimentos, Facultad de Ciencias Biológicas, Universidad Autónoma de Nuevo León, Av. Universidad S/N, San Nicolás de los Garza C.P. 66451, Nuevo León, Mexico; danielaherrerabal@gmail.com (D.D.H.-B.); juan.baezgn@uanl.edu.mx (J.G.B.-G.); numisamaya@hotmail.com (C.A.A.-G.); 2 Laboratorio de Química y Bioquímica, Facultad de Agronomía, Universidad Autónoma de Nuevo León, Francisco Villa S/N, Col. Ex-Hacienda El Canadá, General Escobedo C.P. 66050, Nuevo León, Mexico; mezage@hotmail.com; 3 Laboratorio de Biopolímeros, Centro de Investigación en Alimentación y Desarrollo (CIAD) A.C., Coordinación Hermosillo, Carretera a la Victoria Km 0.6, Hermosillo C.P. 83304, Sonora, Mexico; ecarvajal@ciad.mx; 4 Laboratorio de Química de los Alimentos, Facultad de Salud Pública y Nutrición, Universidad Autónoma de Nuevo León, Av. Dr. Eduardo Aguirre Pequeño y Yuriria, Col. Mitras Centro C.P. 64460, Monterrey, Nuevo León, Mexico; vania.uriaso@uanl.mx

**Keywords:** feruloylated arabinoxylans, nixtamalized maize bran by-product, frankfurter sausages, physicochemical properties, functional properties

## Abstract

Feruloylated arabinoxylans obtained from nixtamalized maize bran were evaluated in terms of physicochemical characteristics and antioxidant capacity when incorporated in frankfurter sausages. Concentrations of 0.15% and 0.30% of feruloylated arabinoxylans were incorporated in frankfurter sausages formulations and a control without feruloylated arabinoxylans was also prepared. Shear force, hardness, color measurement, proximate analysis, pH, titratable acidity, water-holding capacity, total phenols, and antioxidant capacity were evaluated. Phenolic content and antioxidant capacity were significantly higher (*P* < 0.0001) in all treatments, sausages containing feruloylated arabinoxylans compared to the control. The results showed that there was a significant difference (*P* < 0.0001) in total phenolic content and antioxidant capacity with all feruloylated arabinoxylans sausages treatments higher than control. Additionally, significant differences (*P* < 0.0001) were obtained in the physicochemical parameters.

## 1. Introduction

Cooked sausages are a complex mix of different food components, including proteins, salts, gels made from muscular proteins and emulsions that contain stabilized fat. Any type of meat can be used to make cooked sausages and they are commonly consumed either hot or cold. Frankfurters are short and small-diameter sausages, made in a finely chopped form and typically used as appetizers [1,2]. Frankfurter sausages are produced with a high fat content, therefore, it is necessary to use fat replacer ingredients in their production to get a product with less fat content, dietary fiber being a good ingredient for this purpose [3]. Dietary fiber is defined as “the edible parts of plants or analogous carbohydrates that are resistant to digestion and absorption in the human small intestine with complete or partial fermentation in the large intestine. Dietary fiber includes polysaccharides, oligosaccharides, lignin, and associated plant substances. Dietary fibers promote beneficial physiological effects including laxation, and/or blood cholesterol attenuation and/or blood glucose attenuation”. The constituents of dietary fiber are: Analogous carbohydrates lignin substances, lignin complex, and non-starch polysaccharides [4]. In addition, a concept called antioxidant dietary fiber was introduced by Saura-Calixto [5], defining this term as: “dietary fiber rich in associated polyphenol compounds that combines in a single material the physiological effect of both dietary fiber and antioxidants”. 

In the production of tortilla, maize is subject to nixtamalization process in which grains are cooked in lime at temperature higher than 70 °C for a time ranging from 5 to 180 min, followed by a steep/soak in the lime cooking solution from 1 to 24 h and finally the bran is completely removed and considered a by-product. Although bran is partially solubilized during nixtamalization, it still contains a large number of hemicelluloses [6,7]. The traditional use for maize bran by-product is mainly animal feed at a low price, but food applications may also provide commercial and nutritional added value, as it is composed largely by feruloylated arabinoxylans (FAX) [8] that has prebiotic and antioxidant properties in both, in vivo and in vitro studies [9].

Our research group recently reported that FAX extracted from nixtamalized maize bran are synonymous with soluble antioxidant dietary fiber because of 85% of FAX are soluble dietary fiber and they contain, as a part of their chemical structure, ferulic acid that is linked by an ester bound to the arabinoxylan chain. In addition, this soluble antioxidant dietary fiber showed a considerable amount of ferulic acid by HPLC, total phenolics by Folin-Ciocalteu and significant antioxidant capacity levels by ABTS, DDPH, FRAP and ORAC assays [10]. The addition of ferulic acid to foods inhibits lipid peroxidation and subsequent oxidative spoilage. Several other industrial applications are based on the antioxidant potential of ferulic acid [11]. In addition, studies have shown that arabinoxylans have positive effects on human health, such as anti-inflammatory and anti-carcinogenic effects [12]. On the other hand, several studies have evaluated the antioxidant capacity and physicochemical characteristics of frankfurter sausages formulated adding natural extracts [2,3,13,14,15,16,17]. The FAX incorporated in the frankfurter sausage formulation can be related to both their antioxidant activity and physicochemical characteristics. The aim of this research was to evaluate the effect of different concentrations (0.15–0.30%) of FAX in the formulation of frankfurter sausages on physicochemical and nutraceutical properties.

## 2. Results and Discussion

### 2.1. Physicochemical Parameters

The pH, titratable acidity, shear force, and diameter were affected (*P* < 0.0001) by the addition of FAX in the sausage formulation (Table 1). The lowest pH value was found in TC with 5.28 and the treatments with FAX had values around 5.44 and 5.49. Treatment 4 formulated with 0.30% of FAX obtained higher pH value than treatment 3 formulated with 0.15% of FAX. In titratable acidity, T4 and T5 obtained the highest values with 0.51% but not statistically different from T2, T3, and T6, while the TC y T1 showed the lowest with 0.37% and 0.35%, respectively. Water holding capacity was higher in 30% formulation only when the extraction time of FAX was 6 h, whereas for the other extraction times, percentage of fiber did not affect this parameter. On the other hand, all experimental treatments of FAX presented higher water holding capacity than control. The addition of FAX to the formulation of frankfurter sausages produced a significant increase (*P* < 0.05) in hardness, except for T3 and T5 which were not statistically different from the control. Moreover, this increase was especially highlighted as it included 0.30% of FAX. In this regard, the treatments T1, T2, T4, and T6 showed higher hardness than TC, with 30.65 N, 68.29 N, 54.72 N and 59.46 N respectively. In addition, in shear force, the same treatments were higher than control with 5.57 N, 5.97 N, 5.70 N, and 6.53 N, respectively.

All the frankfurter sausages formulated with FAX, except T3 with 63.02%, had a higher water holding capacity and therefore, a larger diameter than control showing a significant difference (*P* < 0.05) (Table 1). These results could be attributed to the fact that the FAX (soluble dietary fiber) holds the free water in the meat product, turning it into bound water [10]. Lower free water content in the product will increase its shelf life due to less oxidation occurring. The pH value has a significant impact on color, shelf life, taste, microbiological stability, yield and texture of meat and meat products. A high pH may cause shorter shelf life, on the other hand, fat and water separation can be caused by low pH [1]. The pH values of meat and meat products are generally between 4.6 (raw fermented salami) and 6.4. At a pH value of around 6.4, meat is spoiled owing to enzyme activity, which produces a large number of metabolic by-products as well as ammonia. The values obtained in this study were 5.28–5.52, which indicates an optimum pH. Regarding hardness and shear force, TC presented the lowest values, but not statistically different from T3 and T5. On the other hand, T2 had the greatest hardness and T6 had the highest shear force but not statistically different from the rest of treatments with FAX.

Schmiele et al. [18] reported a hardness between 50.86–70.36 N by adding dietary fibers (cellulose fiber) as a fat substitute, these results are similar to the values found in treatments T2, T4, and T6 which were formulated with 0.30% FAX. On the contrary, Cáceres et al. [19] used fructooligosaccharides in cooked sausage and found no significant effect on hardness compared with the control treatment.

### 2.2. Color Parameters

There were statistical differences in most of the chromatic properties measured (Table 2). The main statistical differences were observed in L* (*P* = 0.0002), in which the treatments with 0.15% of FAX (T1, T3 and T5) had the values from 75.66 to 80.73. On the contrary, all the treatments with 0.30% of FAX (T2, T4, and T6) obtained values from 68.20 to 70.16, with T2 being statistically different from the control. In a* values, all the treatments were positive (redness) and the main differences were observed among treatments T1 with the higher value but not statistically different from T3, T4 and T5. The treatments TC, T2, and T6 showed lower values (*P* = 0.0088). Also, all b* values were positive (yellowness) and treatment T4 showed a statistical difference (*P* ˂ 0.0001) with all of the treatments. It was observed that the concentration of FAX influenced greatly this behavior. The L* and a* values were higher in treatments with 0.15 (T1, T3, and T5). Contrary to this behavior, the b* values were higher in the formulations that included 0.30% of FAX. 

In addition, C* did not show statistical differences (*P* = 0.4332) among treatments and the values ranged from 15.93 to 17.06, while *h* values showed statistical differences (*P* < 0.0001) among treatments with values ranging from 47.86 (T2) to 32.03 (T1). According to the visual color (view) obtained by ColorHexa software [20], TC and T6 are classified as “grayish red” color, while T2 and T4 are classified as “slightly desaturated red” color and T1, T3, and T5 are classified as “very soft red” color.

According to the color view in Table 2, there is a visual difference between treatments formulated at 0.15% and 0.30% of FAX and this behavior could be attributed to the water holding capacity of FAX, because of higher concentrations of FAX hold more free water in food matrix, which could reduce the lightness of the product. 

Different studies have reported the use of natural sources in the formulation of sausages in order to increase their functional properties. In this regard, Alvárez and Barbut [21] evaluated the addition of inulin (0.15% to 0.60%) and β-Glucan (3% to 6%) of cooked meat batters. They reported that β-Glucan addition did not affect L*, while decreasing a* and increasing b*. This change in the color can result in cooked samples with a characteristic brown color, slightly different from the pink color in the cooked sausages. In addition, Choi et al. [22] investigated the effects of reducing pork fat levels from 30% to 20%, 15%, and 10% by partially substituting pork fat with a makgeolli lees fiber in raw meat batters and frankfurters. They found that the lightness of frankfurters was highest in the control, while the redness and yellowness were similarly increased with increasing makgeolli lees fiber, the obtained values of L* from 75.23 to 84.69, a* from 1.48 to 2.06 and b* from 8.23 to 10.38.

### 2.3. Proximate Analysis

The results of the proximate analysis (moisture, protein, ether extract, crude fiber, and ash) were affected by the addition of FAX (*P* < 0.0001) and are shown in Table 3. The lowest content of moisture was found in TC (50.01%), while treatments T5 (63.13) and T6 (63.08%), in which the FAX extraction time was 6 h, obtained the highest content in this parameter, but not statistically different from T2 and T4. In protein content, there were statistical differences among T1 (15.21%) which obtained the highest in this attribute and the rest of treatments which were in the range of 11.28% (T2) and 13.55 (T6). The fat content was higher in TC, T3 and T5 with 11.38%, 10.32% and 10.93% respectively, while the lower fat content was T1, T2, T4 and T6 with values from 4.88 to 6.08%, the difference among the highest and lowest treatment is close to 50% fat reduction. Méndez-Zamora et al. [3] reported protein and moisture content between 10.24–10.98 and 57.76–61.90, respectively, by adding inulin and pectin as a fat substitute in the formulation of frankfurter sausages. García et al. [23] reported similar changes in fat content. They made sausages by partially replacing the fat content with inulin gels, consequently, they decreased the fat content by up to 44%. Although the molecular weight of FAX was not measured in this work, it is well documented that extraction time under alkaline conditions has an effect on the intrinsic viscosity of FAX which is indicative of their molecular weight, therefore, a long time extraction produced FAX with lower molecular weight [24]. In addition, a long-time extraction also has an effect on the degree of branching, the pattern of branching, constituent monosaccharides and ferulic acid, and these FAX features may affect their behavior in processing [25]. The FAX used in T1 were obtained by 2 h alkaline treatment and showed a 0.82 Ara/Xyl ratio, which is lower than the 0.87 Ara/Xyl ratio of the FAX used in T3 and T5, which were obtained by 4 h and 6 h alkaline treatment, respectively. Also, we hypothesize that T1 had a lower molecular weight than T3 and T5. The different Ara/Xyl ratio and the possible differences in molecular weight of FAX used in these treatments could have produced different interactions in the matrix of sausage, especially in emulsion stability, and maybe because T1 showed lower fat content than T3 and T5. Similar results were found by Mendez-Zamora et al. [3] who used higher fiber concentration (inulin and pectin) and obtained lower fat content. The crude fiber content was lower in TC with 0.18% while the treatments with FAX were statistically different from the TC with values around 0.60 to 0.70%. 

### 2.4. Total Phenolic Content and Antioxidant Capacity

There were statistical differences (*P* < 0.0001) among treatments in total phenolics and antioxidant capacity assays (Table 4). The amount of total phenolics is higher in treatments in which FAX were used with values from 0.060 to 0.073 mgFAE/g compared to TC which showed 0.038 mgFAE/g. Also, the results in antioxidant capacity were higher in DPPH, ABTS, and FRAP for treatments that included FAX in their formulation with values ranging from 0.512 to 0.651, from 0.525 to 0.749 and from 0.140 to 0.176 µmolTE/g, respectively. While the TC obtained was 0.358, 0.412, and 0.069 µmolTE/g DPPH, ABTS and FRAP, respectively. In the DPPH and ABTS assays, T3 and T4, respectively (both obtained by 4 h of alkaline treatment) were not statistically different from the TC. This could be attributed to the fact that the water extracts used for nutraceutical properties assays, contained not only phenolic compounds but also soluble proteins from meat. In the case of TC, the protein is not totally stabilized in the emulsion system of frankfurter sausages as is in treatments containing FAX and probably proteins were extracted in higher quantity in TC. As described by Serpen et al. [26], the meat proteins could act in the radical scavenging in DPPH and ABTS assays, but they have a poor ability to reduce ferric ion to its ferrous form in FRAP assay. 

To the best of our knowledge, this is the first report in which total phenolics and DPPH, ABTS, and FRAP antioxidant capacity assays have been evaluated in frankfurter sausages formulated with FAX. Frankfurter sausages increased their phenolic content and their antioxidant properties due to the addition of FAX finding significant differences among control and treatments, and because of that FAX can be considered as a functional ingredient in this product.

Isaza et al. [27] reported an increase in the antioxidant capacity of the frankfurter sausage evaluating the addition of cherry extract as an ingredient in the formulation, finding the content of total phenolics, ABTS and DPPH values of 0.65 and 0.75 μmolGA /mg, 0.8 and 3.1 μmolTE /g and 0.7 to 1.2 μmolTE /g of sausage, respectively. A study done by Póltorak et al. [28] mixtures of catuaba, galangal and roseroot were added to improve the antioxidant, anti-inflammatory and antimicrobial effects of stored sausages and they reported results of total phenolics ranging from 0.015 to 0.029 μg/mg. Broncano et al. [29] studied the use of proteases to improve the oxidative stability of sausages, however, different methods were used to analyze the antioxidant properties. They reported 58.47–68.11 RSA (radical scavenging activity) evaluated with DPPH.

## 3. Materials and Methods

### 3.1. Feruloylated Arabinoxylans (FAX) Extraction

FAX extraction was carried out according to our previous work [10] and its neutral sugars composition and dietary fiber content is summarized in Table 5. 

### 3.2. Treatments

A completely randomized design of seven treatments was established in this research. Each treatment was the combination of frankfurter sausages plus the concentration of FAX used in different formulations (0.15 and 0.30%) at different extraction times (2 h, 4 h, 6 h) (Table 6). A control was prepared using frankfurter sausage without the addition of FAX.

### 3.3. Preparation of Frankfurter Sausages

The formulation and ingredients used in the preparation of frankfurter sausages were made according to the methods established by Méndez-Zamora et al. and Deda et al. [3,30] with some modifications. The procedure consisted of the following steps: (1) The pork and beef meat were partially thawed and ground for 3 min to then add the nitrites and 1/3 of ice, (2) the polyphosphates were added and another 1/3 ice was incorporated and continued to mill for 2 min, (3) the sausage seasoning was added, and the emulsification process continued for 2 min, keeping the temperature below 11 °C, (4) the partially thawed pork fat was added and ground for 2 min, (5) the starch along with remaining ice was added, and the grinding process continued for 3 min. (6) Once the meat paste was prepared, it was stuffed into cellulose casings (3 cm diameter). The sausages were tied with a thread manually every 15 cm. (7) Sausages were then cooked in a water bath at 80 °C and placed in a polyethylene bag to prevent washing of the ingredients until they reached an internal temperature of 68 °C, (8) finally the sausages were cooled in an ice water bath (4 °C for 20 min) and refrigerated at 4 °C until the analysis. For the preparation of the T1 to T6 treatments, the FAX were added in the fifth step together with the starch used in the formulations.

### 3.4. Hardness and Shear Force (SF)

The hardness and SF were performed using a TA.XT Plus Stable Micro Systems texture analyser (Surrey, UK) according to the method used by Méndez-Zamora et al. [3]. The hardness was performed with three central sections (3.0 cm in height and 2.5 cm diameter) at 4 °C with a 75 mm diameter compression platen to compress the sample using 1.0 mm/s pre-test speed of, 5.0 mm/s test and 5.0 mm/s post-test speed. For SF a Warner–Bratzler blade was fitted into the texturometer, and three central sections of each sample (3.0 cm in height and 2.5 cm in diameter) were used at a temperature of 4 °C for the test. The conditions established for the test were: 2 mm/s pre-test speed, 2 mm/s test speed, 10 mm/s post-test speed and a total distance deformation of 30 mm. 

### 3.5. Color Measurements

Samples evaluated before in harness and shear force were split in two and used for color determination. The inner part of the frankfurter sausages was measured using a CR-20 Konica Minolta Color Reader (Tokyo, Japan). Chromatic parameters were obtained using CIELAB (L*, a*, b*) and CIELCH (L*, C*, *h*) color systems according to Commission Internationale De L’ecleirage [31]. L* defines Lightness (0 = black, 100 = white), a* indicates red (positive a*) or green value (negative a*) and b* indicates yellow (positive b*) or blue value (negative b*), C* (Chroma, saturation level of *h*) and *h* (hue angle: 0° = red, 90° = yellow, 180° = green, 270° = blue). The color view was obtained by online software ColorHexa color [20] converter using L*, a*, and b* values.

### 3.6. Water Holding Capacity (WHC)

The WHC of the sausages was determined using the compression method described by Méndez-Zamora et al., Tsai et al., and Dzudie et al. [3,32,33] with some modifications. Approximately 300 mg of sausage was placed between two filter papers (Whatman 20–25 µm), then placed between two 12 × 12 cm plexiglas plates, and a force of 4.0 kg was applied for 20 min. Due to the force exerted on the sample, released liquids were impregnated in the paper, and it was considered as the free water of the meat. 

### 3.7. pH and Titratable Acidity (TA)

The pH of sausages was measured with an AE150 Fisher Scientific pH meter (Pittsburgh, PA, USA). Two grams of sausage in 20 mL of distilled water was ground in a blender until a homogeneous mixture was obtained. The TA was measured in 10 mL of homogeneous sample, which was previously filtered. Phenolphthalein was added as an indicator and titrated with 0.4 N NaOH. Calculations were performed using the following Equation:

% TA = [(mL NaOH × N NaOH × acid meq)/mL sample] × 100 × DF

where: N = normality of NaOH, acid meq= citric acid meq (0.0064), DF = dilution factor.

### 3.8. Proximate Analysis

Proximate analysis was determined using AOAC methods [34]. Moisture (method 925.09), protein (N × 6.25) (method 960.52), ash (method 923.03), fat (method 923.03), and crude fiber (method 920.86) were evaluated samples. Results were expressed in terms of percentage (g/100 g).

### 3.9. Total Phenolics (TP) and Antioxidant Capacity (AC)

The phenolic extracts of sausages were obtained by the method described by Liu et al. [35]. Extracts were obtained by milling 25 g of sample in 50 mL of distilled water, after that, samples were heated to boiling and left for 20 min, then cooled in a freezer (−4 °C), filtered and refrigerated until use. TP determination and DPPH, ABTS, and FRAP antioxidant capacity assays were performed using the method established by López-Contreras et al. [36]. For TP, 200 μL of extract was taken, 2600 μL of distilled water and 200 μL of the Folin–Ciocalteu reagent were added. After 5 min, 2000 μL of 7% Na_2_CO_3_ was added. The solution was stirred for 30 s, and the reaction was carried out in the dark for 90 min. Finally, the absorbance of the samples was measured at 750 nm. The concentration was obtained using the linear regression equation of the calibration curve established with ferulic acid at concentrations of 0 to 200 mg/L. The results were expressed in milligrams equivalents of ferulic acid per gram of sample (mgFAE/g). DPPH (2,2-Diphenyl-1-picrylhydrazyl) radical test was performed by adding 1500 μL of DPPH to 50 μL of the extract. Absorbance was read at 515 nm after 30 min of reaction in darkness. ABTS assay (2,2′-azinobis (3-ethylbenzothiazol-6-sulfonic acid)), was performed using the stock solution prepared by combining the 7.4 mM ABTS reagent mixture and the 2.6 mM potassium persulfate solution (1:1 *v*/*v*). The solution was diluted in 80% methanol and adjusted to obtain an absorbance of 0.700. Fifty microliters of extract were taken and allowed to react with 1500 μL of ABTS for 30 min in darkness. Absorbance was read at 734 nm. FRAP assay was performed by mixing stock solutions, 300 mM sodium acetate buffer, 10 mM TPTZ (2,4,6-tripyridyltriazine complex) and 20 mM iron chloride hexahydrate in 10:1:1 proportion at a temperature of 37 °C. Samples (50 μL) were reacted with 1500 μL of the FRAP solution for 30 min at 37 °C. The absorbance was read at 593 nm. The concentration was obtained using the linear regression equation of the calibration curve established with Trolox at concentrations of 0 to 500 μmol/L. Results were expressed as micromoles of Trolox equivalents per gram of sample (μmolTE/g) in DPPH, ABTS and FRAP assays. 

### 3.10. Statistical Analysis

The variables evaluated were analyzed by a completely randomized design. The GLM procedure of SAS® [37] was used for the analysis of the data and the following statistical model: yij = μ + Ti + εij, where: yij = response variables, µ = general mean, Ti = treatment effect, εij = random error with mean zero and variance σ2 [εij ~ N (0, σ2)]. A probability of Type I error of less than 0.05 was established to provide the effect of treatments. To compare the means between treatments the Tukey test was performed. All analyses were carried out in triplicate.

## 4. Conclusions

Feruloylated arabinoxylans extracted from nixtamalized maize bran under different alkaline conditions enhanced the physicochemical properties and antioxidant capacity of frankfurter sausages when added to them as an ingredient in the formulation. Extraction time is positively correlated to the hardness, shear force, water holding capacity and color of the frankfurter sausages. Determination of total phenolics and antioxidant capacity is higher in the treatments that included feruloylated arabinoxylans due to the presence of ferulic acid bound by an ester bond to the arabinoses of the xylose chains. This study demonstrated that feruloylated arabinoxylans are polysaccharides that can be incorporated into frankfurter sausages to enhance their physicochemical and nutraceutical properties, and because of that FAX can be considered as a functional ingredient in this product. 

## Figures and Tables

**Table 1:** Physicochemical parameters of frankfurter sausages treatments.

Treatment	Physicochemical Parameter					
	pH	TA (%)	WHC (%)	HA(N)	SF (N)	DIA (mm)
TC (control)	5.28 ^c^	0.37 ^b^	57.03 ^c^	19.38 ^d^	3.36 ^b^	23.13 ^c^
T1	5.44 ^a,b^	0.35 ^b^	64.97 ^b^	30.65 ^c^	5.57 ^a^	25.31 ^b^
T2	5.49 ^a^	0.49 ^a^	69.20 ^a,b^	68.29 ^a^	5.97 ^a^	26.05 ^a,b^
T3	5.41 ^b^	0.46 ^a^	63.02 ^b,c^	23.52 ^c,d^	5.14 ^a,b^	26.35 ^a,b^
T4	5.48 ^a^	0.51 ^a^	70.58 ^a,b^	54.72 ^b^	5.70 ^a^	26.63 ^a^
T5	5.44 ^a,b^	0.51 ^a^	65.24 ^b^	26.23 ^c,d^	4.65 ^a,b^	26.86 ^a^
T6	5.47 ^a,b^	0.48 ^a^	73.58 ^a^	59.46 ^a,b^	6.53 ^a^	26.83 ^a^
SE	0.01	0.01	1.62	2.27	0.40	0.22
*P*-value	<0.0001	<0.0001	<0.0001	<0.0001	0.0030	<0.0001

Different letters within the same column are significantly different (*n* = 3). TA = titratable acidity, WHC = water holding capacity, HA = hardness, SF = shear force, DIA = diameter, SE = standard error.

**Table 2:** Chromatic properties of frankfurter sausages treatments.

Treatment	Chromatic Property					
	L*	a*	b*	C*	*h*	View
TC (control)	75.50 ^a,b,c^	11.66 ^b^	10.83 ^b^	15.93 ^a^	42.13 ^a,b^	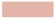
T1	76.23 ^a,b^	14.46 ^a^	9.23 ^b^	17.06 ^a^	32.03 ^c^	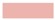
T2	68.20 ^d^	11.60 ^b^	11.46 ^b^	16.50 ^a^	47.86 ^a^	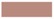
T3	80.73 ^a^	12.53 ^a,b^	10.03 ^b^	16.20 ^a^	38.13 ^b,c^	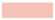
T4	69.03 ^c,d^	12.86 ^a,b^	16.66 ^a^	16.80 ^a^	38.10 ^b,c^	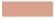
T5	75.66 ^a,b,c^	13.13 ^a,b^	11.23 ^b^	16.93 ^a^	40.63 ^b^	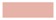
T6	70.16 ^b,c,d^	11.86 ^b^	11.16 ^b^	16.30 ^a^	43.23 ^a,b^	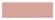
SE	1.41	0.47	0.56	0.40	1.41	---
*P*-value	0.0002	0.0088	<0.0001	0.4332	<0.0001	---

Different letters within the same column are significantly different (*n* = 3). SE = standard error.

**Table 3:** Proximate composition of frankfurter sausages treatments.

Treatment	Component (%)				
	Moisture	Protein	Fat	Crude Fiber	Ash
TC (control)	50.01 ^c^	11.34 ^c^	11.38 ^a^	0.18 ^c^	5.12 ^c^
T1	54.31 ^b,c^	15.21 ^a^	4.88 ^b^	0.72 ^a,b^	5.35 ^b,c^
T2	58.72 ^a,b^	11.28 ^c^	6.08 ^b^	0.78 ^a^	6.63 ^a,b^
T3	54.80 ^b,c^	13.12 ^b^	10.32 ^a^	0.74 ^a,b^	6.25 ^b,c^
T4	59.58 ^a,b^	12.94 ^b^	5.89 ^b^	0.70 ^a,b^	7.62 ^a^
T5	63.13 ^a^	11.37 ^c^	10.93 ^a^	0.70 ^a,b^	6.52 ^b^
T6	63.08 ^a^	13.55 ^b^	5.92 ^b^	0.60 ^b^	6.59 ^a,b^
SE	1.12	0.21	0.50	0.03	0.26
*P*-value	<0.0001	<0.0001	<0.0001	<0.0001	0.0002

Different letters within the same column are significantly different (*n* = 3). SE = standard error.

**Table 4:** Total phenolics and antioxidant capacity of frankfurter sausages treatments.

Treatment	Nutraceutical Property			
	Total Phenols (mgFAE/g)	DPPH (µmolTE/g)	ABTS (µmolTE/g)	FRAP (µmolTE/g)
TC (control)	0.038 ^d^	0.358 ^c^	0.412 ^c^	0.069 ^c^
T1	0.070 ^a,b^	0.512 ^b^	0.665 ^a,b^	0.163 ^a,b^
T2	0.060 ^c^	0.651 ^a^	0.716 ^a,b^	0.153 ^a,b^
T3	0.072 ^a,b^	0.466 ^b,c^	0.749 ^a^	0.140 ^b^
T4	0.073 ^a^	0.641 ^a^	0.568 ^b,c^	0.174 ^a,b^
T5	0.065 ^b,c^	0.637 ^a^	0.657 ^a,b^	0.173 ^a,b^
T6	0.073 ^a^	0.662 ^a^	0.611 ^b^	0.176 ^a^
SE	0.001	0.023	0.033	0.007
*P*-value	<0.0001	<0.0001	<0.0001	<0.0001

Different letters within the same column are significantly different (*n* = 3). SE = standard error.

**Table 5:** Monosaccharide composition, protein content and the dietary fiber content of alkali-extracted feruloylated arabinoxylans from nixtamalized maize bran used in the present study.

FAX	Monosaccharide (%)						Dietary Fiber (%)	
	Xyl	Ara	Gal	Glc	Pro	Ara/Xyl	Sol	Ins
2 h	33.43	27.73	2.52	4.51	1.00	0.82	86.56	ND
4 h	29.69	25.89	2.50	1.98	0.86	0.87	89.95	ND
6 h	30.28	26.64	3.12	1.26	0.62	0.87	86.14	ND

FAX = feruloylated arabinoxylans, Xyl = xylose, Ara = arabinose, Gal = galactose, Glc = glucose, Pro = protein, Sol = soluble, Ins = insoluble, ND = not detected. Adapted from Herrera-Balandrano et al. [10].

**Table 6:** Nomenclature and description of frankfurter sausages treatments.

Treatment Nomenclature	Treatment Description
TC (control)	FS + 0.00% FAX
T1	FS + 0.15% FAX 2 h
T2	FS + 0.30% FAX 2 h
T3	FS + 0.15% FAX 4 h
T4	FS + 0.30% FAX 4 h
T5	FS + 0.15% FAX 6 h
T6	FS + 0.30% FAX 6 h

FS = frankfurter sausage, FAX = feruloylated arabinoxylans, 2 h, 4 h, 6 h = extraction time of FAX.
